# Diferenças Étnicas na Sobrevida entre Medalhistas Olímpicos Brasileiros da Era Moderna de 1920 a 1992: Um Estudo de Coorte

**DOI:** 10.36660/abc.20230524

**Published:** 2024-03-13

**Authors:** Fabricio Braga, Paula de Medeiros, Ana Carolina Neno, Diogo Meira, João Magalhães, Michael S. Emery

**Affiliations:** 1 Laboratório de Performance Humana Rio de Janeiro RJ Brasil Laboratório de Performance Humana, Rio de Janeiro, RJ – Brasil; 2 Casa de Saúde São José Rio de Janeiro RJ Brasil Casa de Saúde São José, Rio de Janeiro, RJ – Brasil; 3 Universidade do Estado do Rio de Janeiro Faculdade de Ciências Médicas Rio de Janeiro RJ Brasil Universidade do Estado do Rio de Janeiro – Faculdade de Ciências Médicas, Rio de Janeiro, RJ – Brasil; 4 Vascular and Thoracic Institute Department of Cardiovascular Medicine Cleveland Ohio EUA Cleveland Clinic – Sports Cardiology Center – Department of Cardiovascular Medicine – Heart, Vascular and Thoracic Institute, Cleveland, Ohio – EUA

**Keywords:** Medicina esportiva, Epidemiologia, Demografia

## Abstract

**Fundamento:**

As disparidades nos resultados de saúde entre grupos raciais merecem investigação, mesmo em atletas de elite. Portanto, compreender o impacto da raça na sobrevida pós-medalha em atletas olímpicos brasileiros torna-se essencial.

**Objetivo:**

Comparar a sobrevida pós-medalha entre medalhistas olímpicos brasileiros brancos e não brancos de 1920 a 1992.

**Métodos:**

Utilizamos dados disponíveis publicamente para um estudo de coorte retrospectivo de todos os medalhistas olímpicos brasileiros de 1920 a 1992 (somente homens). Os atletas foram classificados nos grupos brancos e não brancos usando determinação estruturada de etnia. As análises de Kaplan-Meier calcularam o tempo médio de sobrevida restrito (TMSR) para cada grupo étnico. Uma análise de riscos proporcionais de Cox avaliou as diferenças de sobrevida baseadas na etnia, ajustando para a idade da conquista da medalha e ano de nascimento (p<0,05).

**Resultados:**

Entre 123 atletas (73,9% brancos), a idade média da conquista de medalhas foi de 25,03 ± 4,8 anos. Durante o estudo, 18,7% dos atletas brancos e 37,5% dos atletas não brancos morreram (p=0,031). Os atletas brancos tiveram média de idade ao óbito de 75,10 ± 18,01 anos, enquanto os atletas não brancos tiveram idade média de 67,13 ± 14,90 anos (p=0,109). O TMSR para atletas brancos foi de 51,59 (IC 95%, 49,79 - 53,39) anos, e para atletas não brancos foi de 45,026 (IC 95%, 41,31 - 48,74) anos, resultando em um ΔTMSR de 6,56 (IC 95%, 2,43 - 10,70; p=0,0018). A análise multivariada mostrou que atletas não brancos apresentavam maior risco de mortalidade do que atletas brancos (RC 5,58; IC 95%, 2,18 - 14,31).

**Conclusão:**

Após a primeira medalha, os atletas olímpicos brasileiros brancos normalmente desfrutam de uma expectativa de vida seis anos mais longa do que seus colegas não brancos, ilustrando uma acentuada diferença de mortalidade e disparidades de saúde entre indivíduos saudáveis no Brasil.

**Figure:**
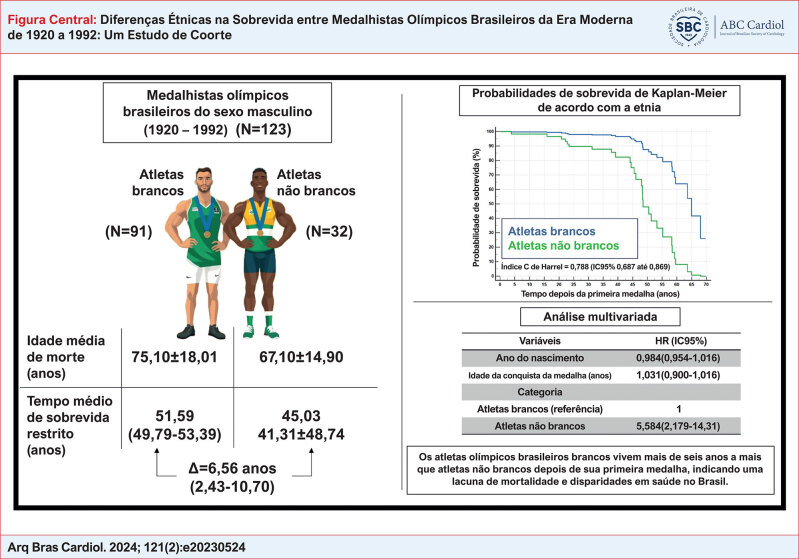


## Introdução

Ser ativo é crucial para reduzir as chances de diversas doenças.^1^ No entanto, têm sido levantadas preocupações em relação aos benefícios para a saúde em situações extremas de exercício,^2^ mais especificamente entre atletas de elite. Apesar dessas preocupações teóricas, a vantagem de longevidade do atleta de elite sobre a população em geral em diferentes países, esportes e etnias, incluindo os medalhistas olímpicos,^3 – 6^ apresentou um aumento da expectativa de vida.^7^

Apesar de um melhor condicionamento físico aumentar a expectativa de vida, infelizmente, as disparidades sociais a reduzem. Por exemplo, existe uma disparidade de longevidade entre pessoas brancas e não brancas, independentemente do nível de desenvolvimento do país.^8 , 9^ Esse fenômeno já foi observado em países desenvolvidos, mesmo entre coortes de atletas.^10^

O Brasil é um país latino-americano de renda média, com uma sociedade multirracial e diversas disparidades sociais que afetam a expectativa de vida.^11^ De acordo com o Instituto de Pesquisa Econômica Aplicada^12^ (instituição pública brasileira voltada para pesquisas socioeconômicas), a expectativa de vida ao nascer era de 73,8, 69,5, 68,2 e 63,2 anos para mulheres brancas, mulheres negras, homens brancos e homens negros, respectivamente. No entanto, essas diferenças são ainda mais proeminentes nas regiões mais pobres.^13^

O efeito de um estilo de vida atlético sobre essas disparidades de longevidade entre brasileiros brancos e não brancos ainda não havia sido considerado até agora. Além disso, apesar do grande número de estudos que abordam a vantagem da longevidade dos atletas de elite, nenhum estudo incluiu atletas de países de renda baixa ou média.

Este estudo tem como objetivo comparar a expectativa de vida dos medalhistas olímpicos brasileiros, distinguindo entre atletas brancos e não brancos, utilizando um banco de dados disponível publicamente.

## Métodos

Todos os dados foram obtidos pelo acesso a informações públicas e não necessitaram de revisão ética, de acordo com os princípios delineados na Declaração de Helsinque de 1975, atualizada em 2013. Seguimos as diretrizes do STROBE para relatar estudos de coorte.

### Definição da coorte de atletas

Todos os medalhistas olímpicos da era moderna entre 1920 (primeira participação da seleção brasileira) e 1992 foram incluídos na coorte de atletas. Esse período foi escolhido para permitir pelo menos 30 anos de acompanhamento. O período de acompanhamento começou quando o atleta conquistou a medalha e terminou com a definição do desfecho, vivo ou falecido. No caso de medalhistas que receberam múltiplas medalhas, foi considerado a primeira.

As informações sobre data de nascimento, óbito e conquista de medalhas dos atletas foram obtidas no site www.olympedia.com,^14^ que contém estatísticas e dados detalhados sobre os atletas olímpicos, apoiado pelo OlyMADMen® e já disponível para uso em pesquisas de sobrevida.^7^ A ausência da data do falecimento no site www.olympedia.com não indicava necessariamente que o atleta estava vivo. Para confirmar, buscamos no site do Comitê Olímpico Brasileiro ( www.cob.org.br ) e na Confederação Brasileira do esporte pelo qual o atleta conquistou a medalha olímpica. Após essa tripla verificação, um atleta foi considerado vivo na ausência de informação de óbito. Os dados sobre o status de vida dos atletas foram recuperados entre 5 e 25 de maio de 2022.

### Definição étnica dos atletas

A definição étnica dos atletas foi realizada pela análise de retratos digitais por três pesquisadores sem qualquer conhecimento das informações biográficas de todos os atletas. Eles receberam um arquivo compactado contendo diversas pastas numeradas, cada uma com cinco a dez fotos do mesmo atleta obtidas na internet. Imagens coloridas foram escolhidas, quando disponíveis. Depois de fazer a análise visual das fotos, eles classificaram o atleta como branco ou não branco (negro, asiático, indígena ou multirracial). Membros de diferentes grupos étnicos analisaram as imagens para reduzir as chances de parcialidade pela própria raça.^15^ Cada um dos pesquisadores também não tinha conhecimento das demais análises. Para fazer a definição étnica, era necessário obter a maioria relativa.

### Envolvimento dos pacientes

Nenhum paciente ou público esteve envolvido na concepção do estudo, nem nos planos de coleta de dados. Não foi pedido a nenhum atleta qualquer orientação sobre a interpretação ou redação dos resultados. Dada a relevância social deste tema, pretendemos divulgar os seus resultados pela mídia em geral após a sua publicação.

### Análise estatística

Devido à natureza exploratória deste estudo, não foi realizado cálculo de tamanho amostral. As variáveis categóricas foram expressas como contagens (n) ou porcentagens (%) e comparadas pelo teste χ2 ou exato de Fisher. As variáveis contínuas foram definidas como média ± desvio padrão e intervalo e foram comparadas por meio do teste t de Student não pareado. A normalidade foi avaliada pelo teste de Kolmogorov-Smirnov.

A concordância na resposta da classificação étnica foi medida pelo coeficiente Kappa de Fleiss.^16^

O TMSR com seu intervalo de confiança (IC) de 95% para cada grupo étnico foi calculado usando análises de Kaplan-Meier considerando o ponto de tempo máximo relatado (54,17 anos).^17^ A análise de sobrevida de riscos proporcionais de Cox foi usada para verificar se as diferenças na sobrevida por etnia eram significativas após o ajuste da idade da medalha e do ano de nascimento.

O nível de significância estatística foi definido como p-valor de <0,05. Todas as análises foram realizadas utilizando o software Statistical Package for the Social Sciences (IBM SPSS® Statistics for Windows, versão 22.0, IBM Corp., Armonk, NY) e MedCalc® Statistical Software, versão 20.110 (MedCalc Software Ltd, Ostend, Bélgica; https://www.medcalc.org ; 2022).

## Resultados

**Figura 1:**
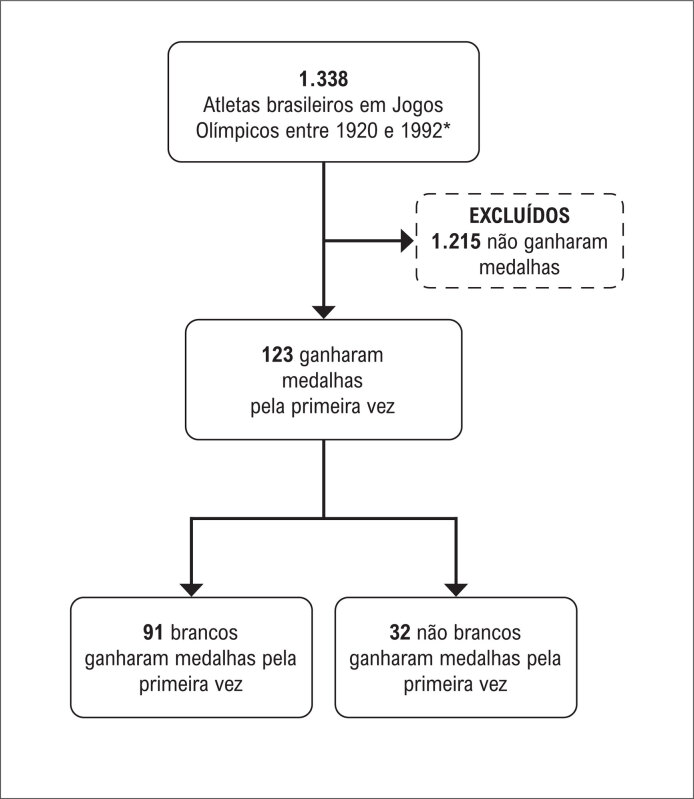
Fluxograma da coorte de atletas olímpicos brasileiros.

A Figura 1 esquematiza a composição da coorte. Entre 1920 e 1992, 1.338 atletas brasileiros participaram de 16 dos 17 Jogos Olímpicos realizados nesse período de 72 anos. Entre eles, 123 (9,2%; todos homens; 25,03 ± 4,8 anos, nascidos entre 1869 e 1972) conquistaram 124 medalhas em doze edições de Olimpíadas. Nove esportes contribuíram para o quadro de medalhas olímpicas brasileiras, contendo 20 medalhas de ouro, 55 de prata e 48 de bronze. Essa pontuação refere-se ao número de atletas premiados, ou seja, nos esportes coletivos, uma medalha representa um atleta. O acompanhamento médio foi de 40,92 ± 11,30 anos, variando de 3,78 a 69,81. A Tabela 1 resume a coorte de atletas.

*Fonte: www.olympedia.com

**Tabela 1:** Resumo da coorte

		Todos (n=123)	Atletas brancos (n=91)	Atletas não brancos (n=32)	p valor
**Idade da conquista da medalha (anos)**		25,03 ± 4,8	24,93 ± 4,8	25,31 ± 4,91	0,703
**Idade da morte**		71,80 ± 17,0	75,10 ± 18,01	67,13 ± 14,90	0,109
**Esporte (n)**					
	Atletismo	6	0	6	
	Basquetebol	26	22	4	
	Boxe	1	0	1	
	Futebol	36	21	15	
	Judô	6	6	0	
	Natação	8	8	0	
	Tiro	5	2	3	
	Vela	12	12	0	
	Voleibol	23	20	3	
**Jogos olímpicos** ( * )					
	Antuérpia 1920	5	2	3	
	Londres 1948	10	9	1	
	Helsinque 1952	3	1	2	
	Roma 1960	12	10	2	
	Tóquio 1964	5	4	1	
	Cidade do México 1968	4	2	2	
	Munique 1972	1	1	0	
	Montreal 1976	2	1	1	
	Moscou 1980	8	8	0	
	Los Angeles 1984	36	30	6	
	Seul 1988	24	12	12	
	Barcelona 1992	13	11	2	
**Tipo de medalha**					
	Ouro	20	15	5	
	Prata	55	38	17	
	Bronze	48	38	10	

* Considerando apenas jogos olímpicos em que o atleta conquistou a primeira medalha.

A técnica de definição étnica adotada identificou 91 (71,9%) atletas brancos e 32 (28,1%) atletas não brancos. O kappa de Fleiss entre os respondentes foi de 0,664 (IC 95%, 0,592 - 0,736), representando uma concordância significativa. A idade média em que atletas brancos e não brancos conquistaram a primeira medalha não diferiu.

Durante o acompanhamento médio de 65,62 ± 8,66 anos, 17 (18,7%) atletas brancos e 12 (37,5%) atletas não brancos morreram (p=0,031; Razão de chance [RC] = 2,61; IC 95% 1,07 a 6,35). A média de idade do óbito foi de 75,10 ± 18,01 e 67,13 ± 14,90 anos (p=0,109) para atletas brancos e não brancos, respectivamente. O TMSR após a conquista da medalha foi de 51,6 (IC 95%, 49,8 a 53,4) e 45,0 (IC 95%, 41,3 a 48,7) anos para atletas brancos e não brancos (ΔTMSR=6,5; IC 95%, 2,4 a 10,7; p=0,0018), respectivamente.

**Figura 2:**
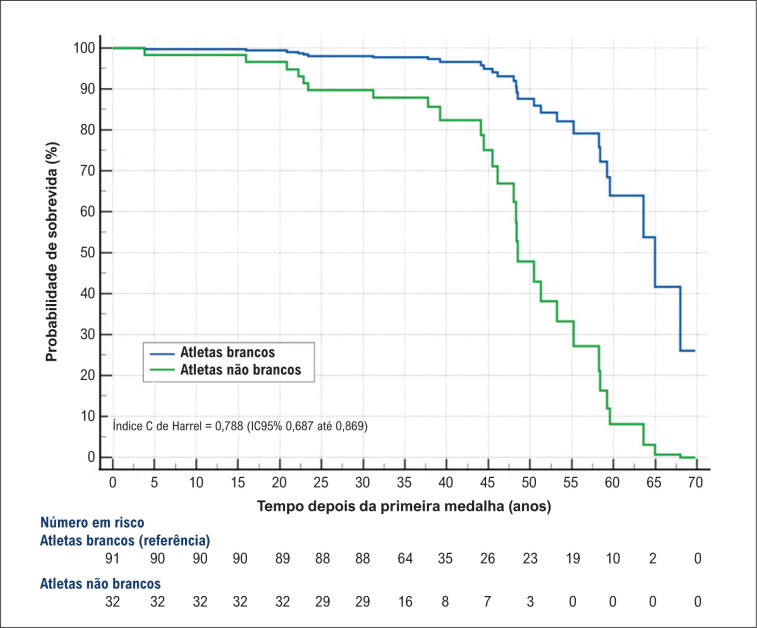
Probabilidades de sobrevida de acordo com a etnia.

A Tabela 2 e a Figura 2 apresentam os resultados de nossa análise multivariada de sobrevida de riscos proporcionais de Cox. Em comparação com atletas brancos, os atletas não brancos exibiram um risco significativamente elevado de mortalidade, com uma taxa de risco (HR) de 5,58 (IC 95%, 2,18-14,31). A Figura Central fornece uma visão geral esquemática do desenho do estudo e de suas conclusões, oferecendo uma representação visual abrangente e integrada.

**Tabela 2:** Modelo de regressão de Cox

Variáveis	β	SE	p valor	HR (IC95%)
Ano do nascimento	-0,016	0,005	0,329	0,984 (0,954-1,016)
Idade da conquista da medalha (anos)	0,031	0,025	0,658	1,031 (0,900-1,016)
Categoria	1,72		<0,001	
Atletas brancos (referência)				1
Atletas não brancos	-1268	0,48	<0,001	5,584 (2,179-14,31)

## Discussão

Até onde sabemos, este é o primeiro estudo que aborda a expectativa de vida dos atletas olímpicos de países em desenvolvimento e como as diferenças étnicas a influenciam.

Depois de conquistarem uma medalha olímpica, os atletas não brancos viveram 6,56 anos menos que os seus colegas brancos. O risco de eles morrerem durante o período do estudo foi mais de cinco vezes maior.

Embora não tenhamos comparado a longevidade dos atletas com a da população em geral, essa lacuna não diferiu daquela observada em estudos brasileiros de disparidades raciais em coortes de não atletas.^13^ De acordo com o último relatório brasileiro sobre desigualdades raciais (2010), a expectativa de vida entre os negros é de 67 anos, contra 73 para os brancos.^18^ Lawler et al.^10^ identificaram que os jogadores brancos do sexo masculino da NBA tinham uma vida útil de 1,5 anos a mais do que os jogadores negros, mas ambos viviam mais do que o público em geral, apesar da cor da pele. Além disso, as diferenças entre as expectativas de vida de negros e brancos foram mais significativas para os não atletas do que para os atletas.

As razões por trás das diferenças na expectativa de vida entre brancos e não brancos nas Olimpíadas brasileiras são multifatoriais. Contudo, provavelmente não diferem daquelas apontadas pelo Instituto Brasileiro de Geografia e Estatística.^19^ As desigualdades no acesso à saúde e à educação e os níveis mais elevados de violência urbana, entre outras disparidades sociais, são responsáveis pela maior vulnerabilidade dos negros brasileiros,^11^ levando à sua menor expectativa de vida mesmo entre os atletas.

Existem muitos determinantes sociais da saúde, uma condição ou circunstância em que as pessoas nascem, crescem, vivem, trabalham e envelhecem, que é moldada por motivações políticas, sociais e econômicas.^20^ Assim, um fator regional pode influenciar o impacto da condição social, tornando-a mais ou menos relevante localmente como determinante da saúde. O racismo estrutural tem sido identificado como um preditor essencial de saúde na sociedade brasileira. Hone et al.,^21^ analisando mais de três milhões de indivíduos em atenção primária à saúde, mostraram que a raça/etnia negra foi um preditor independente de multimorbidade, definida pelo diagnóstico de duas ou mais entre 53 condições crônicas (RC: 1,05; IC 95%: 1,03, 1,06; comparado aos brancos). Além disso, a probabilidade de morte em cinco anos também foi maior entre os negros (1,48% [IC 95%: 1,41, 1,55%] comparado a 1,35% [IC 95%: 1,31, 1,40%] para brancos).

Algumas limitações merecem consideração neste estudo. Em primeiro lugar, a nossa categorização de atletas em brancos e não brancos com base na análise de fotografias pode introduzir possíveis erros de classificação. No entanto, dada a ausência de dados oficiais sobre a etnia dos atletas, especialmente para atletas olímpicos históricos, consideramos esse método uma alternativa razoável. Além disso, tomamos precauções para aumentar a precisão desta análise; com exceção de cinco atletas da década de 1920, a etnia de todos os outros atletas foi determinada pela avaliação de fotografias coloridas por um painel diversificado, reduzindo o risco de parcialidade pela própria raça. Da mesma forma, a distribuição dos esportes entre atletas brancos e não brancos (conforme mostrado na Tabela 1 ) alinha-se com vieses raciais documentados nas equipes olímpicas (por exemplo, nenhum medalhista branco de atletismo e nenhum medalhista não branco de natação).^22^ Em segundo lugar, embora tenhamos tentado incluir todos os medalhistas olímpicos brasileiros sem perda de acompanhamento, o tamanho da nossa amostra permaneceu limitado, uma vez que alcançar uma inclusão abrangente de todos os atletas olímpicos brasileiros ainda é um trabalho em andamento. Em terceiro lugar, esta coorte não inclui atletas mulheres, pois foi apenas em 1996 que uma brasileira conquistou uma medalha olímpica. Em quarto lugar, é importante reconhecer que ser medalhista olímpico não garante uma saúde superior em comparação com a população em geral, apesar de numerosos estudos sugerirem tal tendência.^23^ Este estudo centra-se nas disparidades de expectativa de vida entre os medalhistas olímpicos com base na raça e, embora apresente informações valiosas sobre esse aspecto específico, não trata de forma abrangente o estado geral de saúde dos atletas olímpicos em relação à população em geral. Em quinto lugar, não conduzimos uma comparação direta da expectativa de vida dos atletas com a da população em geral, o que poderia fornecer informações valiosas sobre as implicações do sucesso olímpico para a saúde. Por último, é importante reconhecer que não conseguimos identificar as causas de morte de todos os atletas da coorte de nosso estudo. Muitos dos atletas do nosso grupo faleceram antes da Segunda Guerra Mundial, e as certidões de óbito deles não estão prontamente disponíveis.

## Conclusão

Esses achados ressaltam uma disparidade significativa na expectativa de vida pós-Olimpíada entre medalhistas brancos e medalhistas não brancos brasileiros. Os medalhistas brancos tiveram uma vantagem na expectativa de vida de mais de seis anos depois de atingirem o auge do sucesso esportivo. Embora o nosso estudo destaque estas disparidades, é importante reconhecer que os benefícios para a saúde associados aos medalhistas olímpicos não eliminam as disparidades na saúde resultantes das desigualdades sociais. No entanto, é essencial reconhecer que, embora ser um atleta olímpico ofereça certas vantagens para a saúde, fatores sociais mais amplos continuam a desempenhar um papel significativo na determinação dos desfechos globais de saúde. Mais pesquisas sobre a relação multifacetada entre raça, atletismo e saúde são necessárias para se chegar a uma compreensão mais abrangente dessas dinâmicas.

N’ *A Ilíada* , Homero apresenta o dilema do semideus mitológico Aquiles de escolher entre uma vida curta e gloriosa como guerreiro e uma existência longa e obscura como cidadão grego comum.^24^ No estudo de Clarke et al.,^7^ os autores fizeram a analogia de que antes dos benefícios de sobrevida dos atletas olímpicos, eles poderiam aproveitar ambos os destinos. Infelizmente, não podemos garantir esse precioso presente aos heróis olímpicos não brancos brasileiros.
