# Supplementary material for: Diferenças Étnicas na Sobrevida entre Medalhistas Olímpicos Brasileiros da Era Moderna de 1920 a 1992: Um Estudo de Coorte
Source: Arq Bras Cardiol. 2024 Mar 13;121(2):e20230524. [Article in Portuguese] doi: 10.36660/abc.20230524 (PMC12092026; doi:10.36660/abc.20230524)
Supplement: Translation

# Translation: Ethnic Differences in Survival among Brazilian Modern-era Olympic Medalists from 1920 to 1992: A Cohort Study

**Authors:** Fabricio Braga, Paula de Medeiros, Ana Carolina Neno, Diogo Meira, João Magalhães, Michael S. Emery

**Affiliations:** 1 Rio de Janeiro RJ Brazil Laboratório de Performance Humana, Rio de Janeiro, RJ – Brazil; 2 Rio de Janeiro RJ Brazil Casa de Saúde São José, Rio de Janeiro, RJ – Brazil; 3 Rio de Janeiro RJ Brazil Universidade do Estado do Rio de Janeiro – Faculdade de Ciências Médicas, Rio de Janeiro, RJ – Brazil; 4 Cleveland Ohio USA Cleveland Clinic – Sports Cardiology Center – Department of Cardiovascular Medicine – Heart, Vascular and Thoracic Institute, Cleveland, Ohio – USA

**Keywords:** Sports Medicine, Epidemiology, Demography

## Abstract

**Background:**

Disparities in health outcomes among racial groups warrant investigation, even among elite athletes. Therefore, understanding the impact of race upon post-medal survival in Brazilian Olympians becomes essential.

**Objective:**

To compare post-medal survival between white and non-white Brazilian Olympic medalists from 1920 to 1992.

**Methods:**

This study used publicly available data for a retrospective cohort study on all Brazilian Olympic medalists from 1920 to 1992 (males only). Athletes were classified into white and non-white groups using structured ethnicity determination. Kaplan–Meier analyses computed the restricted mean survival time (RMST) for each ethnic group. A Cox proportional hazards analysis assessed ethnicity-based survival differences, adjusting for medal-winning age and birth year (p<0.05)

**Results:**

Among 123 athletes (73.9% white), the mean age of medal achievement was 25.03±4.8 years. During the study, 18.7% of white and 37.5% of non-white athletes died (p=0.031). White athletes had a mean age at death of 75.10±18.01 years, while non-white athletes had an age of 67.13±14.90 years (p=0.109). The RMST for white athletes was 51.59 (95% CI 49.79-53.39) years, while for non-white athletes, it was 45.026 (95% CI 41.31-48.74) years, resulting in a ΔRMST of 6.56 (95% CI 2.43-10.70; p=0.0018). Multivariate analysis showed that non-white athletes had a higher mortality risk than did white athletes (HR 5.58; 95% CI, 2.18-14.31).

**Conclusion:**

Following their first medal, white Brazilian Olympians typically enjoy a six-year longer lifespan than their non-white counterparts, illustrating a marked mortality gap and health disparities among healthy individuals in Brazil.

## Introduction

Being active is crucial in reducing the odds of several diseases.^1^However, concerns have been raised regarding the health benefits in extreme exercise situations,^2^more specifically among elite athletes. Despite these theoretical concerns, the elite-level athlete's longevity advantage throughout the general population in different countries, sports, and ethnicities,^3 – 6^including Olympic medalists, has shown an increased lifespan.^7^

As better fitness improves life expectancy, unfortunately, social disparities reduce it. For example, there is a longevity gap between white and non-white people, regardless of the country's level of development.^8 , 9^This phenomenon has already been observed in developed countries, even among athletic cohorts.^10^

Brazil is a Latin American middle-income country with a multiracial society and several social disparities affecting life expectancy.^11^According to the Institute of Applied Economic Research^12^(a Brazilian public institution addressed socioeconomic research), life expectancy at birth was 73.8, 69.5, 68.2, and 63.2 years for white women, black women, white men, and black men, respectively. However, those differences are even more prominent in the poorest regions.^13^

The effect of an athletic lifestyle on these longevity disparities between white and non-white Brazilians has, to date, never been considered. Moreover, despite the large number of studies addressing the lifespan advantage of elite athletes, no single study has included athletes from low- or middle-income countries.

This study aims to compare the life expectancy of Brazilian Olympic medalists, distinguishing between white and non-white athletes, using a publicly available database.

## Methods

All data were obtained by accessing public information and did not require ethical review, in accordance with the principles outlined in the 1975 Helsinki Declaration, updated in 2013. Our study followed the STROBE guidelines for reporting cohort studies.

### Athletic cohort definition

All modern-age Olympic medalists between 1920 (first Brazilian team participation) and 1992 were included in the athletic cohort. This time frame was chosen to allow at least 30 years of follow-up. The follow-up period started when the athlete gained the medal and finished with the endpoint definition, alive or deceased. In the case of multiple medalists, the first was considered.

Athletes’ information regarding date of birth, death, and medal achievement was obtained at www.olympedia.com,^14^a website containing statistics and detailed data about Olympians, supported by OlyMADMen® and already available for use in survival research.^7^The absence of a death date at www.olympedia.com did not necessarily indicate that the athlete was alive. As such, we searched the Brazilian Olympic committee website ( www.cob.org.br ) and the Brazilian sports confederation, for which the athlete won the Olympic medal. After this triple check, an athlete was considered alive in the absence of information about his death. The data on the athletes’ life status were retrieved between May 5^th^and May 25^th^, 2022.

### Athlete's ethnic definition

The athletes’ ethnic definition was performed via digital portrait analysis by three researchers blinded to all athletes’ biographic information. They received a compressed file containing several numbered folders, each with five to ten photos of the same athlete obtained on the internet. Colored images were preferred when available. After visually analyzing the photos, they classified the athlete as white or non-white (black, Asian, indigenous, or multiracial). Members of different ethnic groups analyzed the images to reduce the odds of own-race bias.^15^Each researcher was also blinded to the other's analysis. A relative majority was required for ethnic definition.

### Patient involvement

No patients or the public were involved in designing the study, nor were they involved in data collection plans. No athlete was asked to advise on interpretation or write-up of the results. Given the social relevance of this topic, we intend to disseminate its results by general media results after its publication.

### Statistical analysis

Because of the exploratory nature of this study, no sample size calculation was performed. Categorical variables were expressed as counts (n) or percentages (%) and compared by χ2 or Fisher's exact test. Continuous variables are defined as the mean±standard deviation and range, and were compared using the unpaired Student's t-test. Normality was assessed using the Kolmogorov-Smirnov test.

The agreement on ethnic classification response was measured using the Fleiss Kappa coefficient.^16^

The restricted mean survival time (RMST) with its 95% confidence interval (CI) for each ethnic group was calculated using Kaplan–Meier analyses, considering the maximum time point reported (54.17 years).^17^Cox proportional hazard survival analysis was used to ascertain whether differences in survival by ethnicity were significant after adjusting for medal-winning age and birth year.

The statistical significance level was defined as a p-value<0.05. All analyses were performed using the Statistical Package for the Social Sciences software (IBM SPSS® Statistics for Windows, version 22.0, IBM Corp., Armonk, NY) and MedCalc® Statistical Software, version 20.110 (MedCalc Software Ltd, Ostend, Belgium; https://www.medcalc.org ; 2022)

## Results

Figure 1 schematizes the cohort composition. Between 1920 and 1992, 1,338 Brazilian athletes participated in 16 of the 17 Olympic Games held in these 72 years. Among them, 123 (9.2%; all men; 25.03±4.8 years, born between 1869 and 1972) achieved 124 medals in 12 Olympics. Nine sports contributed to the Brazilian Olympic medal table, containing 20 gold, 55 silver, and 48 bronze medals. This score refers to the number of prized athletes, i.e., for team sports, one medal represents one athlete. The mean follow-up was 40.92±11.30 years, ranging from 3.78 to 69.81. Table 1 summarizes the athletic cohort.

**Figure 1:** Flowchart of the Brazilian Olympians cohort.

*Source: www.olympedia.com

**Table 1:** Cohort summary

		All (n=123)	White athletes (n=91)	Non-white athletes (n=32)	p value
**Medal-winning age (years)**		25.03 ± 4.8	24.93 ± 4.8	25.31 ± 4.91	0.703
**Death age**		71.80 ± 17.0	75.10 ± 18.01	67.13 ± 14.90	0.109
**Sport (n)**					
	Athletics	6	0	6	
	Basketball	26	22	4	
	Boxing	1	0	1	
	Football	36	21	15	
	Judo	6	6	0	
	Swimming	8	8	0	
	Shooting	5	2	3	
	Sailing	12	12	0	
	Volleyball	23	20	3	
**Olympic games** ( * )					
	Antwerp 1920	5	2	3	
	London 1948	10	9	1	
	Helsinki 1952	3	1	2	
	Roma 1960	12	10	2	
	Tokyo 1964	5	4	1	
	Mexico City 1968	4	2	2	
	Munich 1972	1	1	0	
	Montreal 1976	2	1	1	
	Moscow 1980	8	8	0	
	Los Angeles 1984	36	30	6	
	Seul 1988	24	12	12	
	Barcelona 1992	13	11	2	
**Medal type**					
	Gold	20	15	5	
	Silver	55	38	17	
	Bronze	48	38	10	

* Considering only Olympic games where an athlete conquered the first medal

The ethnic definition technique adopted in this study identified 91 (71.9%) white and 32 (28.1%) non-white athletes. The Fleiss kappa between responders was 0.664 (95% CI 0.592-0.736), representing substantial agreement. The mean age at which white and non-white athletes won their first medal did not differ.

During the mean follow-up of 65.62±8.66 years, 17 (18.7%) white and 12 (37.5%) non-white athletes died (p=0.031; Odds Ratio [OR]=2,61; 95% CI 1.07 to 6.35). The mean age of death was 75.10±18.01 and 67.13±14.90 years (p=0.109) for white and non-white athletes, respectively. The RMST after medal win was 51.6 (95% CI 49.8 to 53.4) and 45.0 (95% CI 41.3 to 48.7) years for white and non-white athletes (ΔRMST=6.5; 95% CI 2.4 to 10.7; p=0.0018), respectively.

Table 2 and Figure 2 present the outcomes of our multivariate Cox proportional hazard survival analysis. In comparison to white athletes, non-white athletes exhibited a significantly elevated risk of mortality, with a hazard ratio (HR) of 5.58 (95% CI, 2.18-14.31). The Central Illustration provides a schematic overview of both the study's design and its findings, offering a comprehensive and integrated visual representation.

**Table 2:** Cox regression model

Variables	β	SE	p-value	HR (95%CI)
Year of birth	-0.016	0.005	0.329	0.984 (0.954-1.016)
Medal-winning age (years)	0.031	0.025	0.658	1.031 (0.900-1.016)
Category	1.72		<0.001	
White athletes (reference)				1
Non-white athletes	-1268	0.48	<0.001	5.584 (2.179-14.31)

**Figure 2:** Survival probabilities according to the ethnicity.

## Discussion

To the best of our knowledge, this is the first study of its kind to address the life expectancy of developing world Olympians and how ethnic differences influence this.

After conquering an Olympic medal, non-white athletes lived 6.56 years less than their white counterparts. Their hazard of dying throughout the study period was more than five times higher.

Although we did not compare athletes’ longevity with the general population, this gap did not differ from that observed in Brazilian racial disparities studies in non-athletic cohorts.^13^According to the last Brazilian report on racial inequalities (2010), the life expectancy among black people was 67 years, as compared to 73 years for whites.^18^Lawler et al.^10^identified that white male NBA players enjoyed a lifespan of 1.5 years more than black players, but both lived longer than the general public, despite their skin color. Moreover, black and white lifetime differences were more significant for non-athletes than for athletes.

The reasons behind the differences in life expectancy between white and non-white Brazilian Olympians are multifactorial. However, they probably do not differ from those highlighted by the Brazilian Institute of Geography and Statistics.^19^Health and education access inequalities and higher levels of urban violence, among other social disparities, account for the higher vulnerability of black Brazilians,^11^leading to their lower lifespan even among the athletes.

There are many social determinants of health, a condition or circumstance in which people are born, grow, live, work, and age, which is shaped by political, social, and economic drives.^20^Thus, a regional factor can influence the impact of the social condition, making it more or less locally relevant as a health determinant. Structural racism has been identified as an essential health predictor in Brazilian society. Hone et al.,^21^analyzing more than three million individuals concerning primary health care showed that black race/ethnicity was an independent predictor of multimorbidity, defined by the diagnosis of two or more out of 53 chronic conditions (OR: 1.05; 95% CI: 1.03,1.06; compared to white). Moreover, the five-year probability of death was also higher among black individuals (1.48% [95%CI: 1.41,1.55%] when compared to 1.35% [95%CI: 1.31,1.40%] for whites).

Some limitations warrant consideration in this study. First, our categorization of athletes into white and non-white based on photograph analysis may introduce potential misclassifications. Nevertheless, given the absence of official data on athletes’ ethnicity, especially for historical Olympians, we deemed this method a reasonable alternative. Furthermore, we took precautions to enhance the accuracy of this analysis; with the exception of five athletes from the 1920s, the ethnicity of all other athletes was determined through the evaluation of colored photographs by a diverse panel, reducing the risk of own-race bias. Moreover, the distribution of sports among white and non-white athletes (as shown in Table 1 ) aligns with documented racial biases within Olympic teams (e.g., no white track and field medalists and no non-white swim medalists).^22^Second, although we endeavored to include all Brazilian Olympic medalists without loss of follow-up, our sample size remained limited, as achieving a comprehensive inclusion of all Brazilian Olympians remains a work in progress. Third, this cohort does not include female athletes, as it was only in 1996 that a Brazilian woman secured an Olympic medal. Fourth, it is important to acknowledge that being an Olympic medalist does not guarantee superior health when compared to the general population, despite numerous studies suggesting such a trend.^23^Our study focuses on life expectancy disparities among Olympic medalists based on race, and while it provides valuable insights into this specific aspect, it does not comprehensively address the overall health status of Olympic athletes in relation to the broader population. Fifth, we did not conduct a direct comparison of athletes’ lifespans with those of the general population, which could provide valuable insights into the health implications of Olympic success. Finally, it's important to acknowledge that we were unable to identify the causes of death for all athletes in our study cohort. Many of the athletes in our cohort passed away before the Second World War, and death their certificates are not readily available.

## Conclusion

These findings underscore a significant disparity in post-Olympic life expectancy between Brazilian white medalists and non-white medalists. White medalists enjoyed a life expectancy advantage of more than six years after reaching the pinnacle of sporting success. While our study highlights these disparities, it is important to acknowledge that the health benefits associated with Olympic medalists do not eliminate the health disparities resulting from social inequities. However, it's crucial to recognize that while being an Olympian offers certain health advantages, broader social factors continue to play a significant role in determining overall health outcomes. Further research into the multifaceted relationship between race, athleticism, and health is warranted in order to gain a more comprehensive understanding of these dynamics.

In *The Iliad* , Homer introduces the dilemma of the mythological demigod Achilles to choose between a short and glorious life as a warrior and a long and obscure existence as a regular Greek citizen.^24^In the study by Clark et al.,^7^the authors made the analogy that before the Olympians’ survival benefits, they could profit from both destinies. Unfortunately, we cannot guarantee this precious gift for the Brazilian non-white Olympic heroes.
